# An Introduction to Traditional and Novel Alternative Proteins for Low- and Middle-Income Countries

**DOI:** 10.1016/j.cdnut.2023.102014

**Published:** 2023-10-11

**Authors:** Jacquelyn R. Bedsaul-Fryer, Jimena Monroy-Gomez, Kesso G. van Zutphen-Küffer, Klaus Kraemer

**Affiliations:** 1 Sight and Life, Basel, Switzerland; 2 Department of Human Nutrition & Health, Wageningen University & Research, Wageningen, The Netherlands; 3 Johns Hopkins Bloomberg School of Public Health, Baltimore, MD, United States

## Landscape of Alternative Proteins for Low- and Middle-Income Countries

Undernutrition remains a major public health issue in low- and middle-income countries (LMICs), particularly as it relates to protein intake [1]. Currently, diets in LMICs are mainly composed of cereals and legumes, while total protein, including animal protein, consumption is much lower than in high-income countries (HICs) [[2], [3], [4], [5]]. However, animal-sourced protein consumption in LMICs is expected to increase in the coming years as incomes rise and populations grow [6,7]. By 2050, over 9 billion people will need adequate, affordable diets to fulfill their macro- and micronutrient needs. Meanwhile, the current food system is responsible for roughly 30% of total greenhouse gas emissions [8,9]. Given that climate change is projected to cause massive consequences to the planet and impact the whole food value chain [9], it is essential to consider diverse food sources to achieve local, sustainable food production, mitigate climate change, and reduce all forms of malnutrition in LMICs and worldwide [1].

**FIGURE 1:**
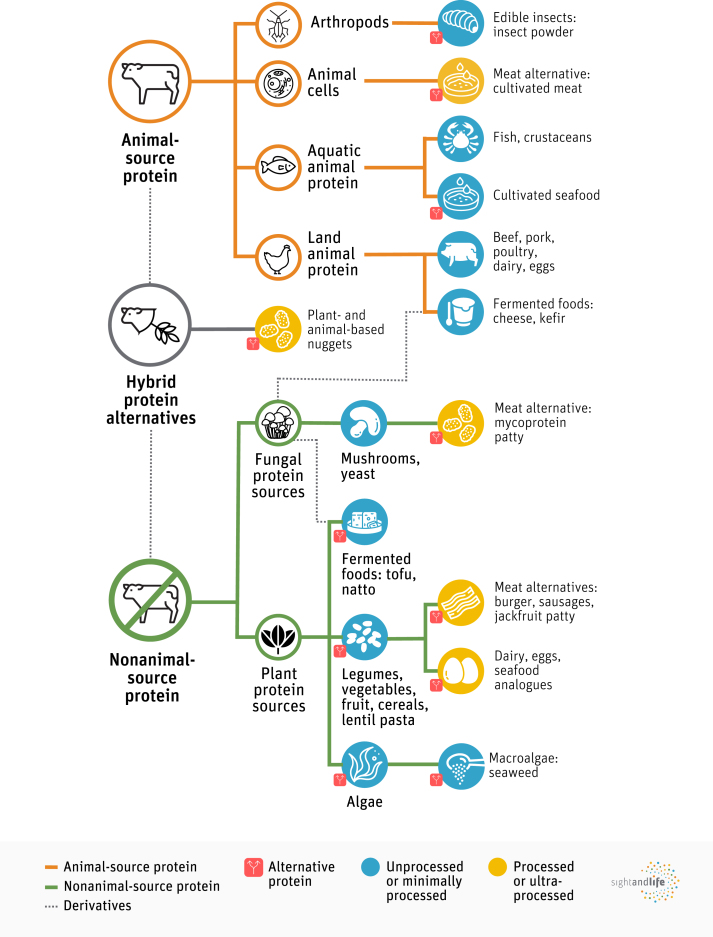
Sources of protein for human food consumption. Both animal (orange line) and nonanimal (green line) sources of protein are used to generate alternative proteins (red symbol). Derivatives (gray line) include hybrid protein alternatives such as plant- and animal-based products. Fermented foods are derivatives of both animal and nonanimal sources of protein and utilize microbes such as yeasts. The processing levels required to generate the diverse protein products are considered: unprocessed or minimally processed (blue fill) and processed or ultraprocessed (yellow fill).

Many foods provide protein in the diet, and protein can come from animal and nonanimal sources (Figure 1). Alternatives to livestock proteins have been positioned as having the potential for positive impact on both people and the planet and to revert the current trajectory of the food system toward sustainability and resilience. However, their ability to provide viable solutions to help address the nutritional (energy, protein, and micronutrient intake), environmental, and economic challenges facing LMICs warrants further exploration and clarity.

The implications of consuming certain protein sources over others are complex and multifaceted, and they vary depending on the factors considered. These variations may involve nuanced factors such as nutritional value, environmental impact, ethics, culture, and personal preferences. Analyzing these aspects of diverse protein sources for different regions is the basis for this supplement entitled “Nourishing 9 billion people by 2050: The role of alternative proteins in low-and-middle-income countries.” This supplement aims to shed light on the latest developments across the alternative protein (AP) sector over the last few decades and draw on the learnings and implications for LMICs. Sharing knowledge and practices related to AP research, production, and integration into the food system and market may encourage the safe development of sustainable, affordable, desirable, and nutritious solutions to improve dietary patterns in LMICs while improving planetary health.

## Terminology Standardization

A critical first step in analyzing diverse protein sources is to standardize the terminology. Various terms are used interchangeably, and often create confusion for consumers and stakeholders alike (e.g., plant-based meat, meat alternative, imitation meat, cultivated meat, plant-based protein, AP, etc.). The lack of clear alignment on terminology also confounds the establishment of standards for literature review, data analysis, guidelines, regulations, and communications, and may delay the development of a framework to galvanize efforts to better evaluate benefits and risks associated with different protein sources. Considering these challenges, we provided a glossary of key terms as an attempt to harmonize concepts and provide clarity on the similarities and differences between them (Table 1) [[10], [11], [12], [13], [14], [15], [16], [17], [18], [19], [20], [21], [22], [23], [24], [25], [26], [27]].

**TABLE 1:** Key terms in the alternative protein field and their definitions

Key terms	Definition
Protein	Macronutrient composed of amino acids that are required for human health and cellular structure and function [10].
Traditional protein	Animal and nonanimal-sourced protein-rich foods that have long been integrated in the diets of specific cultures and regions [11].
Alternative protein	Any protein-rich ingredient sourced from plants, fungi, algae, insects, or animal cells intended to replace conventional livestock products (beef, poultry, pork, fish, seafood, eggs, or dairy) [12,13].
Meat alternative	Also referred to as plant-based meat and meat substitutes, analogs, and surrogates, also denoted as fake, faux, mock, and imitation meat. These products can be nonanimal-sourced or derived from animal cells as opposed to using conventional livestock as ingredients. Processing techniques yield similar sensorial characteristics (texture, flavor, and appearance) compared to animal protein, such as beef, poultry, and pork products [14].
Fermented protein products	Foods or beverages produced through controlled microbial growth, and the conversion of food substrates through enzymatic action (e.g., conversion of phenolic compounds to biologically active compounds). Products are invaded or overgrown by edible microorganisms whose enzymes, particularly amylases, proteases, and lipases, hydrolyze the polysaccharides, proteins, and lipids into nontoxic products with different profiles of nutrients, flavors, aromas, and textures. [15,16]. Examples include tofu, natto, cheese, and kefir.
Animal-sourced protein^1^	Protein from edible insects, aquatic animals, and land animals including meat, eggs, and dairy products [11].
Edible insects	A source of high-quality protein from arthropods (i.e., ants, caterpillars, crickets, grasshoppers, and mealworms) that provide amino acids, fiber, micronutrients, and bioactive components [17,18].
Cultivated meat	Refers to edible protein obtained by collection of cells from living animals and proliferating the cells into meat in vitro using cell engineering, termed cellular agriculture. Cultivated meats for beef, pork, poultry, and seafood are being developed for taste and aesthetic appeal to mimic traditional animal source foods. Also known as lab-grown and cultured meat [19].
Nonanimal-sourced protein^1^	Food or ingredients that provide amino acids from plants and fungi, including vegetables, fruits, mushrooms, yeasts, legumes, nuts, seeds, and cereals [20].
Fungal protein source	Proteins derived from a kingdom independent from plant and animal that consists of mushrooms and truffles (*Basidiomycetes)*, molds, and yeasts, including fungal species used in fermentation processes e.g., *Fusarium venenatum* (*Ascomycetes*), a filamentous fungus that produces ‘mycoprotein’ [21,22].
Mycoprotein	A source of high-quality protein and fiber with a meat-like texture made from *Fusarium venenatum*, a naturally occurring fungus, via fermentation. The production strain was discovered in the 1960s and mycoprotein approved for sale as food protein for the first time in the United Kingdom in 1984 [21].
Plant protein source	Food or ingredient that provide amino acids which are present in or isolated from any part of a plant such vegetables, fruits, pulses, nuts, seeds, and cereal grains [23].
Plant-based protein product	Refers to processed or texturized proteins from leguminous crops (i.e., soybeans, mung and fava beans, chickpeas, peas, and peanuts) and mushrooms, yeast, vegetables, fruits (jackfruit), nuts, or grains (i.e., corn, oats, quinoa, rice, sorghum, wheat, and almonds) that have been used to develop traditional and novel alternatives to address consumer health and/or environmental concerns about producing and consuming animal source proteins [24].
Algae	Includes multicellular organism (macroalgae) such as seaweed and single-celled microorganisms (microalgae) such as spirulina are aquatic animal alternative protein sources with diverse nutritional profiles and bioactive compounds [25].
Hybrid protein alternative	Products that use animal and nonanimal sources of protein to produce a blended alternative protein product. Recent innovations have used peas or soy as nonanimal sources of protein and insects, cultivated, or conventional meat as animal protein source [26,27].

1 Categories of protein sources

Both animal and nonanimal protein sources have long been consumed in different cultures and regions to different extents. Some proteins are traditional in certain cultures, while they may be considered novel alternatives in others. For instance, fermented foods from soy have been traditionally consumed in Asian regions as a protein-rich food source but have only recently been recognized as an alternative to animal-sourced protein in Western regions [28]. To the contrary, insects have been consumed widely in Latin America and Asia but are considered novel protein sources in Europe [11]. Therefore, we reason that the "novel*"* or *"*traditional" connotation depends on cultural–regional practices. In addition, we support the definition of AP to be any protein-rich ingredient sourced from plants, fungi, algae, insects, or animal cells intended to replace conventional livestock products [12]. Figure 1 depicts the relationship between the many terms and protein sources that are described throughout this supplement.

## Processing Considerations for Alternative Proteins

We used the NOVA classification system to help evaluate APs according to the level of processing required to generate the diverse AP products. As shown in Figure 1, unprocessed and minimally processed as well as processed and ultraprocessed AP products exist; these range from plant-based whole foods and lentil pasta to meat alternatives, respectively [29]. Importantly, we recognize that unprocessed and minimally processed plant protein sources can cause digestion and nutrient absorption issues without processing [14,30]. In addition, while plant protein sources are associated with healthy, sustainable diets, they are typically lower in total protein content and essential amino acids compared to animal-sourced protein. Therefore, we may not wish to categorize or critique processed AP products in the same manner as junk food, as certain processing techniques may provide improved nutrient profiles by making proteins and micronutrients more bioavailable, all the while mimicking the taste and mouthfeel of conventional animal-sourced proteins like meat and dairy. Still, ultraprocessed foods are known to contain less desirable nutrient profiles and ingredients that have been associated with noncommunicable diseases [31]. Thus, careful consideration of AP nutritional content and safety are needed.

## The Science and Technology of Alternative Proteins

Within the AP industry, plant protein sources like legumes and cereals are used to generate plant-based protein products through processing techniques that can convert nonanimal proteins into products with meat-like textures. Processed fungal protein sources like mushrooms and yeast can generate novel AP products such as mycoprotein patties that mimic chicken and have comparable protein content, flavors, aromas, and textures to the animal counterpart [21]. On the other hand, cellular agriculture involves culturing animal cells in bioreactors to generate cultivated meat [19]. Other products on the market combine animal- and nonanimal-sourced proteins into a single product, referred to as a hybrid protein alternative (Table 1). Each protein source and product have a role to play in the global food system and require critical evaluation that considers the local context.

## Supplement Composition

This supplement includes articles that analyze the role of APs from 4 distinct aspects, each with a focus on LMICs. First, the authors explore nutritional considerations for APs, including the nutritional needs and challenges of LMIC populations. The nutritional quality and factors regarding AP digestibility, absorption, and bioavailability are discussed. The authors consider the relevance of utilizing local protein sources amidst LMIC protein transitions, as well as the nutritional policies required for developing and marketing APs safely, effectively, and accessibly. The latter topic is still under discussion in HICs; in addition to drawing upon learnings from HIC, is essential to consider various determinants such as culture, preferences, nutrient profiles, and availability of protein sources in LMICs to design context-specific policies.

The supplement also analyzes business models and technology of APs. The authors discuss market opportunities and technological and economic challenges to establish desirable, affordable, and nutritious APs for LMICs. Access to technology and machinery to manufacture processed APs is frequently unavailable or unaffordable for some countries. The authors also examine the consumer perspective through case studies in LMICs, which analyze the opportunities and risks of generating demand for APs and nutritious products. Consumer behavior and acceptance are highlighted as crucial elements for changing dietary patterns and marketing diverse protein sources.

The final part of this supplement explores perspectives on metrics and tools to assess APs from a sustainability angle. Widespread claims about the potential of APs to reduce environmental impacts have been made for certain AP products; however, the metrics used to date have not combined nutritional and environmental measures holistically, which represents a more relevant approach for assessing implications for human and planetary health. Finally, the authors draw attention to the traditional methods of producing food by analyzing diverse AP based on production methods, food processing levels, and their ability to fit with established agroecological principles.

## Closing Remarks

Novel AP products are considered premium items with inaccessible prices in many LMICs today. Moreover, local and culturally appropriate protein sources are not fully harnessed as a sustainable and affordable solution across regions. The articles within this supplement examine whether the current "traditional" and "novel" AP landscape might change for LMICs and what factors catalyze this change. Can the development of AP inputs, infrastructure, demand, and consumption close major nutritional gaps at an affordable price while mitigating climate change? This work represents a step toward acknowledging this possibility and analyzing the necessary considerations for LMIC communities.

We hope that this series of articles inspires entrepreneurs, academics, scientists, and professionals in business, civil society, and government to promote the incorporation of diverse protein sources and further develop evidence-based, safe, affordable, sustainable, and nutritious diets in LMICs. We hope that consumers and stakeholders can learn from the current AP landscape and production practices of this sector, innovate technologies, and implement necessary standards and regulations. Such regulations should include clear communication guidelines around AP production, marketing, and consumption.

Based on the analysis from the many contributors of varied expertise involved in this supplement, we do not claim for or against novel APs for LMICs. Rather, we argue that diverse protein alternatives to conventional livestock sources are needed, and we advocate for additional research and development to fully understand their implications for human and planetary health. It will be essential to engage in systematic thinking and encourage collaboration among diverse stakeholders to analyze and safely integrate emerging novel proteins in LMICs while continuing to promote local and traditional protein sources.

## Author contributions

The authors’ responsibilities were as follows—all authors: conceptualized the paper; JRB-F, JM-G: contributed to the literature review and wrote the first draft of the manuscript; KGvZ-K, KK: provided input, critical review, and shaped subsequent drafts; KK: had primary responsibility for final content; and all authors: read and approved the final manuscript.

## Conflict of interest

JRB-F previously participated in the Alternative Protein Project at Johns Hopkins University that collaborates with the Good Food Institute. Participation was voluntary and no financial support was received. *Sight and Life* funded the special supplement. JM-G, KGvZ-K, and KK report no conflicts of interest related to the topic of this article.

## Funding

The authors reported no funding received for this study.
